# Mucinous colorectal adenocarcinoma: clinical pathology and treatment options

**DOI:** 10.1186/s40880-019-0361-0

**Published:** 2019-03-29

**Authors:** Cong Luo, Shuyi Cen, Guojun Ding, Wei Wu

**Affiliations:** 1 Department of Abdominal Oncology, Zhejiang Cancer Hospital, Hangzhou, 310022 Zhejiang P. R. China; 2 School of Medicine, Zhejiang University, Hangzhou, 310058 Zhejiang P. R. China; 3 Department of Radiotherapy, Zhejiang Cancer Hospital, Hangzhou, 310022 Zhejiang P. R. China; 4 Department of Pathology, Zhejiang Cancer Hospital, Hangzhou, 310022 Zhejiang P. R. China

**Keywords:** Adenocarcinoma, Mucinous carcinoma, Colorectal cancer, MUC2, MUC5AC, Microsatellite instability, Lynch syndrome, Targeted molecular therapy, Hyperthermic intraperitoneal chemotherapy, Immunotherapy

## Abstract

Mucinous colorectal adenocarcinoma is a distinct subtype of colorectal cancer (CRC) characterized by the presence of abundant extracellular mucin which accounts for at least 50% of the tumor volume. Mucinous colorectal adenocarcinoma is found in 10%–20% of CRC patients and occurs more commonly in female and younger patients. Moreover, mucinous colorectal adenocarcinoma is more frequently located in the proximal colon and diagnosed at an advanced stage. Based on its molecular context, mucinous colorectal adenocarcinoma is associated with the overexpression of mucin 2 (MUC2) and mucin 5AC (MUC5AC) proteins. At the same time, it shows higher mutation rates in the fundamental genes of the RAS/MAPK and PI3K/Akt/mTOR pathways. Mucinous colorectal adenocarcinoma also shows higher rates of microsatellite instability (MSI) than non-mucinous colorectal adenocarcinoma which might correlate it with Lynch syndrome and the CpG island methylator phenotype. The prognosis of mucinous colorectal adenocarcinoma as to non-mucinous colorectal adenocarcinoma is debatable. Further, the impaired responses of mucinous colorectal adenocarcinoma to palliative or adjuvant chemotherapy warrant more studies to be performed for a specialized treatment for these patients. In this review, we discuss the molecular background and histopathology of mucinous colorectal adenocarcinoma, and provide an update on its prognosis and therapeutics from recent literatures.

## Introduction

Colorectal cancer (CRC) is one of the leading causes of cancer-related death worldwide [1]. The improvement in individualized treatments calls for refinement of the subtypes’ classification of cancers based on their histological characteristics and genetic features. The most common histologic subtype of CRC is adenocarcinoma, of which mucinous adenocarcinoma is a distinct subtype and is characterized by abundant mucinous components that comprise of at least 50% of the tumor volume [2]. Statistics suggest that 10%–20% of CRC patients are of the mucinous subtype [3, 4], but this rate has been observed to be lower in Asian countries and higher in Western countries [5–8]. In regard to the clinical pathology, mucinous colorectal adenocarcinoma is found more frequently in the proximal colon than in the rectal or distal colon [4, 9, 10]. The ratios of female and younger patients with mucinous colorectal adenocarcinoma are both higher compared to non-mucinous colorectal adenocarcinoma [11–13]. Moreover, mucinous colorectal adenocarcinomas are more frequently diagnosed when they are already in advanced stages and they usually have poorer responses to chemotherapies as compared to their non-mucinous counterparts [14, 15].

Molecular assessments have revealed significant differences between mucinous and non-mucinous colorectal adenocarcinoma, suggesting a different mechanism of oncogenesis. Overexpression of the MUC2 protein is one of the most evident molecular aberrations that distinct mucinous colorectal adenocarcinoma from its non-mucinous counterpart [16–19]. Mucinous colorectal adenocarcinoma is also associated with a high frequency of microsatellite instability (MSI-H), which correlates with Lynch syndrome [5] and mutations that pass through the Ras-Raf-MEK-ERK pathway (RAS/MAPK pathway) [18]. However, the factors involved in the development of the mucinous colorectal adenocarcinoma and their prognostic implications are not yet well understood.

Conflicting results are found in the literatures regarding the prognosis and overall survival (OS) of mucinous colorectal adenocarcinoma patients. Patients with mucinous colorectal adenocarcinoma currently receive treatments based on the same standard guidelines as for CRC. However, considering their impaired response to current chemotherapies, treatments specialized for patients with mucinous colorectal adenocarcinoma histology are urgently needed. In this review, we aimed at discussing the molecular background and histopathology of mucinous colorectal adenocarcinoma, and to evaluate the effects of different treatments provided to these patients at different stages.

## Molecular background of mucinous colorectal adenocarcinoma

Compared with the non-mucinous subtype, mucinous colorectal adenocarcinoma is characterized by a higher ratio of lymph node infiltration and peritoneal implant, often occurring in the proximal colon, and has a significantly larger maximal size [20, 21].

Further, there is a persisting debate in regard to the prognosis of mucinous colorectal adenocarcinoma as compared to its non-mucinous counterpart. Some studies have found that patients with mucinous colorectal adenocarcinoma have a lower progression-free survival (PFS) rate (3-year PFS rate, 79.2% vs. 56.9%, respectively) and a shorter median OS (60.2 months vs. 48.4 months, respectively) [22, 23]. However, others have shown that the mucinous colorectal adenocarcinoma histology itself had no correlation with prognosis. For instance, a population-based analysis consisting of over 120,000 colon cancer patients in Europe demonstrated that mucinous colorectal adenocarcinoma histology had no negative impact on survival [24]. In contrast, Japanese investigators found that mucinous colorectal adenocarcinoma was associated with poorer survival compared to non-mucinous for patients with stage III and IV diseases [25]. Hugen et al. [9] suggested that poor prognosis for mucinous carcinoma only existed in rectal cancer but not in colon cancer. Furthermore, a meta-analysis consisting of 44 studies comprising of over 220,000 patients indicated a poorer prognosis for patients with mucinous colorectal adenocarcinoma histology when the stage at presentation was adjusted [7]. Till present, the prognostic value of mucinous colorectal adenocarcinoma remains undetermined when the locations of the tumor, molecular alterations, population characteristics, or different treatment plans are taken into account. However, it is worth noting that signet-ring cell carcinoma, another subtype of adenocarcinoma characterized by abundant intracellular mucin such that their nucleus is displaced aside, has shared molecular features with mucinous colorectal adenocarcinoma, including the presence of MSI-H, CpG island methylator phenotype-high (CIMP-H), and frequent *BRAF* mutations [26]. It has been reported that compared with colorectal adenocarcinoma and mucinous colorectal adenocarcinoma, patients with signet-ring cell carcinoma were more frequently associated with metastatic disease, metastases at multiple sites, and had a poorer survival than mucinous colorectal adenocarcinoma patients [27–29]. A study by Inamura et al. [26] showed that even when less than 50% component of signet-ring cells were present in the tumor, they could still serve as a poor prognostic indicator in CRC, independent of other clinicopathological features. Several molecular alterations as described below, are related to the occurrence and prognoses of mucinous colorectal tumors.

### MUC2 and MUC5AC expression in mucinous colorectal adenocarcinoma

The human mucin family consists of secreted mucins (such as mucin2 [MUC2], MUC5AC, MUC5B, and MUC6) and transmembrane mucins (such as MUC1, MUC4, MUC13, and MUC16). Mucins form a mucous barrier to protect the epithelia under normal conditions. Epithelial cells of the gastrointestinal tract usually synthesize more than one type of mucin, but the expression of one particular type of mucin may predominate in one specific organ. For instance, MUC2 is more commonly observed in the goblet cells of the small and large intestinal mucosa while MUC6 is predominately found in the gastric epithelium as compared to normal colon [30]. During oncogenesis, the expression of specific mucins may decrease or may even lead to a loss of organ specificity, while the new mucins are aberrantly expressed. The aberrant expression of the mucins is paradoxically associated with inflammation and epithelial cancers. For example, the upregulation of MUC1 was found in response to chronic inflammation while the overexpression of other transmembrane mucins contributes to oncogenesis through the promotion of receptor tyrosine kinase signaling, loss of epithelial cell polarity, constitutive activation of growth and survival pathways, and downregulation of stress-induced death pathways [31]. Mutational analysis of mucin genes performed on five major cancers have revealed an unequal incidence of mutations throughout cancer-associated mucins [32].

It was discovered that *MUC2* and *MUC5AC*, two of the secreted mucin-encoded genes in a cluster on chromosome 11p15.3, correlate with the occurrence of mucinous colorectal carcinoma [33]. Mucinous colorectal adenocarcinoma is associated with higher positivity rate of *MUC2* which produces mucin-2 (MUC2), a secreted protein that functions in the physiological processes of the gastrointestinal tract as a physical protection barrier [16, 18, 31, 34]. MUC2 is predominantly found in the colorectal goblet cells and proximal colon [17, 34]. Observations on the extent of disease in patients have found that the MUC2 expression is associated with the extent of ulcerative colitis and increases the risk of colon cancer [31]. While mucin has recently been used as a target for molecular therapy, the overexpression of MUC2 could form a mucous layer that protects itself against antitumor immune factors, and thus promoting the development of tumors [35, 36]. However, studies in the literature have also indicated that MUC2 could suppress inflammation and inhibit the development of intestinal tumors, and that the loss of MUC2 expression was a predictor of adverse outcomes [30, 31]. The contradictory role of MUC2 as an inflammation suppressor and a promoter for tumor initiation in gastrointestinal tract cancers might suggest that gastrointestinal tract cancers originate from cells that express MUC2 rather than MUC2 itself as playing a role in the malignant process [31].

Another secreted mucin, MUC5AC, which is encoded by the *MUC5AC* gene, is mainly expressed in gastric and tracheal-bronchial mucosa but shows overexpression in over half of the cases of CRC [16]. Clinical studies have shown that the absence of MUC5AC expression could serve as an indicator of a more aggressive colorectal tumor and that patients with MUC5AC-negative expression had lower survival rates [37]. A study in 2008 concluded that SRY (sex determining region Y)-box 2 (SOX2) was important in the upregulation of MUC5AC in mucinous colorectal adenocarcinoma [38] while Raghoebir et al. [39] showed that the aberrant expression of SOX2 did not correlate with mucinous colorectal adenocarcinoma histology or MUC5AC expression. To date, the oncogenic mechanisms underlying MUC2 and MUC5AC have not yet been determined. However, concerning the differences between the gene expression levels in mucinous and non-mucinous colorectal adenocarcinomas, recent literatures suggest that MUC2 and MUC5AC could serve as potential targets for future treatments for mucinous colorectal adenocarcinoma.

### Microsatellite instability (MSI)

MSI is also a critical factor that contributes to the pathology and prognosis of mucinous colorectal adenocarcinoma. Compared to non-mucinous colorectal adenocarcinoma, mucinous colorectal adenocarcinoma is associated with MSI-H [4, 18]. The development of about 70% of CRC cases is associated with chromosomal instability and MSI is detected in the other 15% CRC cases. Among the 15% CRC cases, 12% are sporadic cancers while 3% are caused by Lynch syndrome, an inherited colorectal disorder that increases the risk for many cancers [40]. Hugen et al. [5] concluded that 22%–40% of cases of Lynch syndrome-related CRC were mucinous colorectal adenocarcinoma, suggesting a close relationship between Lynch syndrome and the mucinous occurrence in CRC. It has been reported that mucinous colorectal adenocarcinoma patients with MSI have better survival rates than those with microsatellite stability (MSS) since MSI-H is associated with a decreased risk of metastasis to either the regional lymph nodes or distant organs in CRC as compared to low-frequency microsatellite instability (MSI-L), and similar results were found in terms of recurrence rates [41]. A long-term study by Andrici et al. [42] suggested that the 5-year OS of mucinous MSI/mismatch repair (MMR)-deficient CRC was similar to non-mucinous low-grade colorectal adenocarcinoma. They also found that patients with mucinous MSI/MMR-deficient colorectal adenocarcinoma had significantly better survival rates than non-mucinous high-grade colorectal adenocarcinoma patients (5-year OS, 73% vs. 53%, respectively, *P* < 0.001) or mucinous MSS/MMR-proficient colorectal adenocarcinoma patients (5-year OS, 73% vs. 57%, respectively, *P* = 0.023) [42].

There are three possible molecular changes that have been identified to result in MSI. First, a deficient mismatch repair (MMR) system that result from mutations in MMR genes (MutL homolog 1 [*MLH1*]), MutS protein homolog 2 [*MSH2*], MutS protein homolog 6 [*MSH6*], *PMS2*) could lead to MSI. Impaired DNA MMR leads to genetic hypermutability and the presence of MSI, thus bring about tumors that grow through MMR/MSI, such as Lynch syndrome-correlated CRC [43, 44].

Second, MSI can occur in sporadic CRC with CIMP, which is a molecular subgroup of CRC and the CpG islands are often located in the promoter regions of many tumor suppressors, including *MLH1* [45]. Hypermethylation of the promoter CpG islands, which leads to the silencing of several tumor suppressor genes, may prohibit the suppression of potential oncogenesis [46]. Promoter hypermethylation of *MLH1* commonly occurs in CRC with CIMP since the CIMP group encompassed almost all cases of sporadic MSI cancers [40, 45].

The third molecular change that results in MSI involves mutations in the fundamental genes of the RAS/MAPK pathway, such as B-Raf proto-oncogene (*BRAF*) and v-Ki-ras2 (*KRAS*) mutations. *BRAF* and *KRAS* are both components of the RAS/MAPK pathway, and activation of this pathway promotes cell division and reduces cell apoptosis [45]. Compared with wild-type *KRAS* carcinomas, *KRAS*-mutated tumors were more frequently located in proximal colon and have shown an increased frequency in mucinous differentiation [47–49]. Tumors with high mucin production have greater frequencies (65%) of *KRAS* mutation than tumors without mucin production [48]. Further, approximately 75% of *BRAF* mutation tumors contain a mucinous adenocarcinoma histology, which is more frequent than *BRAF* wild-type and *KRAS/BRAF* wild-type tumors. It has also been reported that *BRAF* mutated patients were associated with adverse histological features and had a significantly shorter median OS of 11.0 months compared to 40.6 months for *BRAF* wild-type patients [49–51]. Recent researches have focused on *BRAF* V600E for its role in MSI status and its prognostic implication. The *BRAF* V600E mutation is the most common *BRAF* mutation, of which accounts for approximately 90% cases and it is reported to be associated with features such as proximal location, MSI, and mucinous adenocarcinoma components [52]. Moreover, the *BRAF* V600E mutation is strongly associated with sporadic origin in MSI-H cases and poor prognosis in advanced CRC [52, 53]. Thus, alterations in the fundamental genes of RAS/MAPK pathway could lead to MSI and are more likely to associate with mucin production, proximal location, and mucinous adenocarcinoma histology than wild-type patients.

### Vascular endothelial growth factor (VEGF)

Early in the year 1995, Takahashi et al. [54] determined that VEGF is a critical angiogenetic factor in primary and metastatic CRC, and may be used as a prognostic factor for predicting metastasis risk in colon cancer. VEGF is frequently expressed in poorly differentiated or mucinous adenocarcinoma of CRC. However, the expression of VEGF as a prognostic factor has been debatable. Ochs et al. [55] suggested that the overexpression of VEGF was associated with poorer survival, while Berk et al. [56] observed an improved PFS and OS in patients with high VEGF expression. In this regard, antiangiogenic treatments deserve further research.

VEGF-targeted therapy has been used more widely in patients with mucinous colorectal adenocarcinoma. Bevacizumab combined with chemotherapy is commonly used as first or second line therapy, and regorafenib or fruquintinib monotherapy is used as third line treatment.

## Imaging of mucinous colorectal adenocarcinoma

**Fig. 1:**
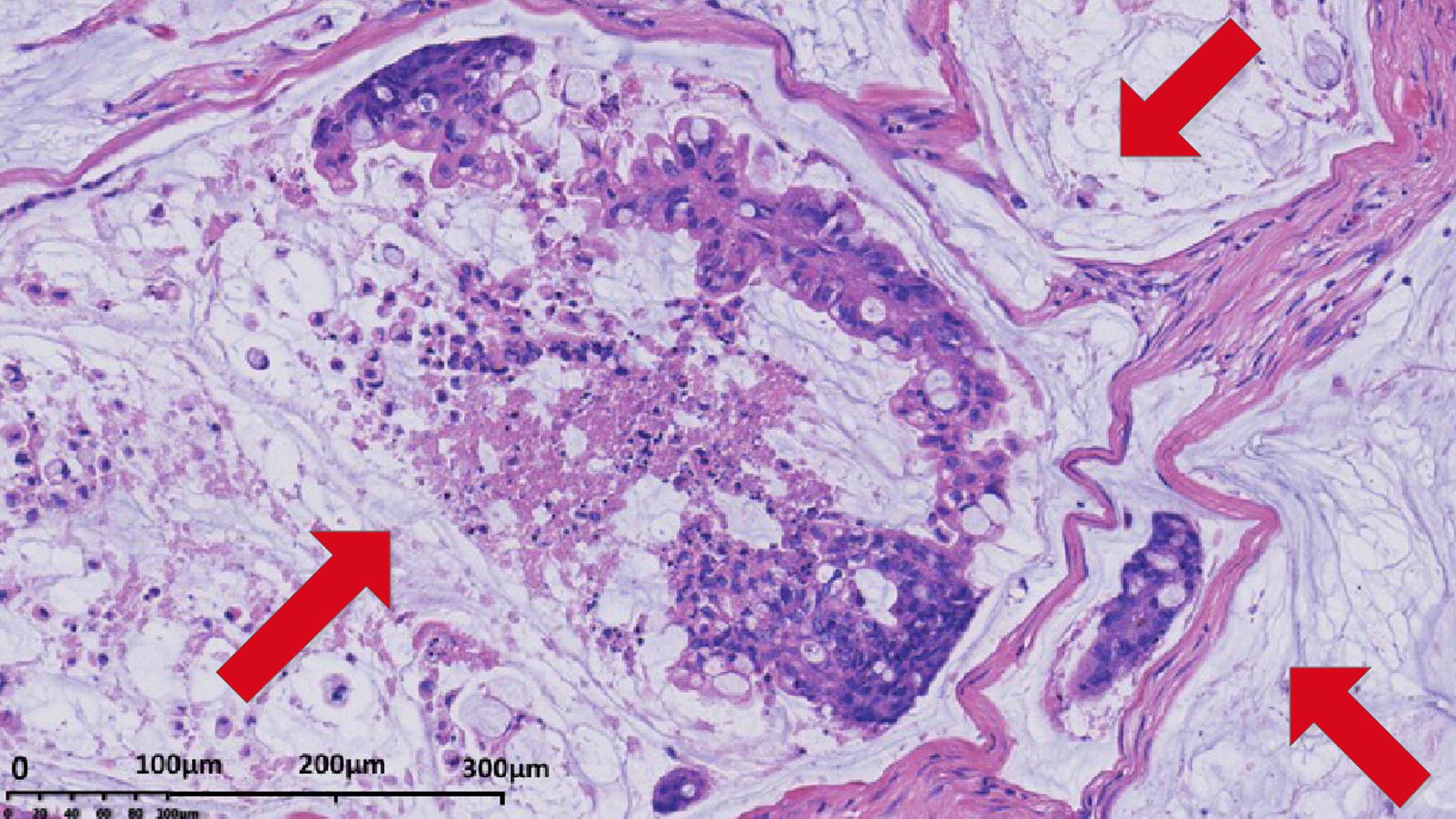
Histopathology of mucinous colorectal adenocarcinoma. H&E stained tissue section from a 53-year-old female patient initially diagnosed with mucinous colorectal adenocarcinoma showing abundant extracellular mucin (red arrows) within the tumor complex. Original magnification, ×20

**Fig. 2:**
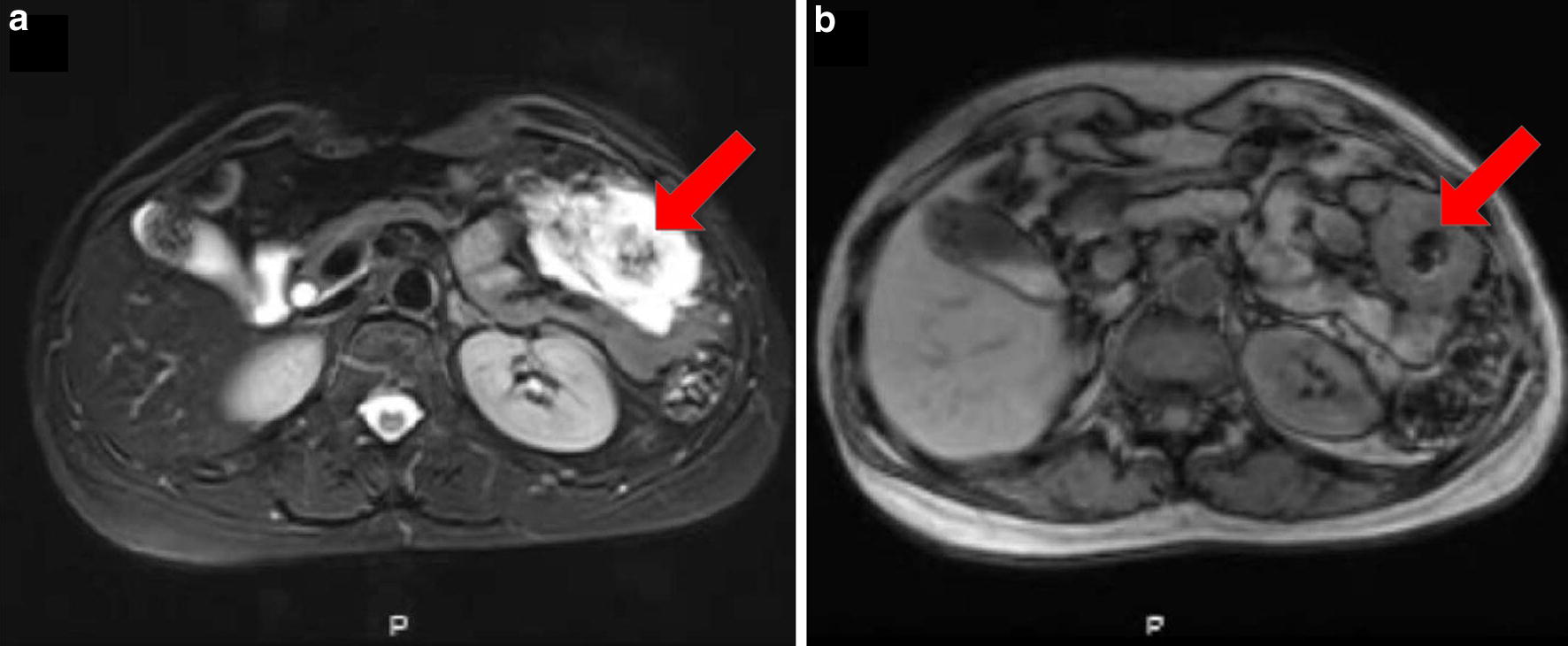
Magnetic resonance images showed an ulcerative mucinous colorectal adenocarcinoma (5.5 × 3.0 × 2.5 cm) located in the transverse colon 55 cm from the anus in a 53-year-old female patient. The patient was diagnosed as mucinous colorectal adenocarcinoma staged T4N2M1 with liver and abdominal metastases. She received 3 cycles of XELOX (capecitabine plus oxaliplatin) chemotherapy, a palliative surgery, 5 cycles of XELOX chemotherapy and capecitabine maintenance therapy for 5 months till present. **a** The axial T2-weighted imaging showed a significantly more intense signal on mucin pools (red arrow) than normal muscle. **b** In the axial T1-weighted image, the mucinous component (red arrow) showed similar signal intensity as to normal muscle

**Fig. 3:**
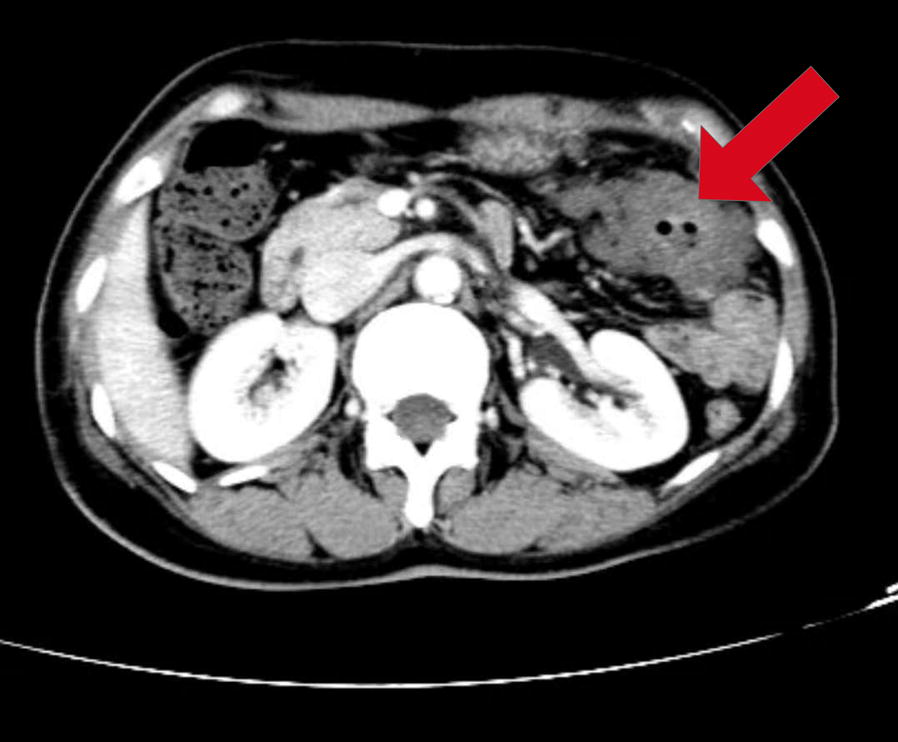
Computer tomography image obtained from a 53-year-old female patient with a mucinous colorectal adenocarcinoma (5.5 × 3.0 × 2.5 cm) located in the transverse colon 55 cm from the anus. No significant enhancement compared with normal muscle in the arterial phase was observed. The patient was diagnosed as mucinous colorectal adenocarcinoma staged T4N2M1 with liver and abdominal metastases. She received 3 cycles of XELOX (capecitabine plus oxaliplatin) chemotherapy, a palliative surgery, 5 cycles of XELOX chemotherapy, and capecitabine maintenance therapy for 5 months till present

Due to the abundant amount of extracellular mucin, mucinous colorectal adenocarcinoma can be distinguished from non-mucinous colorectal adenocarcinoma under light microscopy (Fig. 1). Further, magnetic resonance imaging (MRI) and computed tomography (CT) are other two common means that are frequently used for diagnosing various subtypes of carcinoma. MRI is helpful for detecting mucinous adenocarcinoma components and it is commonly used for differential diagnosis. Mucinous colorectal adenocarcinoma is characterized by a low signal intensity in T1-weighted images and a significant hyperintense signal in T2-weighted images (Fig. 2), while non-mucinous colorectal adenocarcinoma displays an intermediate signal intensity in T2-weighted images [57, 58]. CT is also used for the diagnosis. Mucinous colorectal adenocarcinoma are distinguished by a thickened intestinal wall, a thickened gut mucosa and low-density cystic lesions (Fig. 3), and the non-significant enhancement in arterial phase, compared with normal muscle, is one of the characteristics of mucinous colorectal adenocarcinoma on CT scan [59].

## Comparison of the current treatments and prognosis for mucinous and non-mucinous colorectal adenocarcinoma

Multiple clinical trials have been performed to evaluate the prognostic value of mucinous colorectal adenocarcinoma histology. Although mucinous colorectal adenocarcinoma is different from non-mucinous colorectal adenocarcinoma in terms of gene expression and histology, the standard treatments for colorectal adenocarcinoma are recommended to mucinous colorectal adenocarcinoma patients since no clinical guidelines have been developed specifically for this group of patients. It was reported that patients with mucinous colorectal adenocarcinoma was less responsive to neoadjuvant and adjuvant chemotherapy as compared to those with non-mucinous colorectal adenocarcinoma, due to the mucinous colorectal adenocarcinoma histology [60–62]. As elaborated, mucinous colorectal adenocarcinoma patients had lower OS rates when they received the same therapies as non-mucinous colorectal adenocarcinoma patients. Consequently, specialized treatment plans for patients with mucinous colorectal adenocarcinoma histology are necessary.

Since mucinous colorectal adenocarcinoma is more commonly diagnosed at a more advanced stage, clinical studies on the treatments of mucinous colorectal adenocarcinoma mainly focused on stage II, stage III, and stage IV. Table 1 summarizes 14 clinical studies which compared the median OS and survival rates of mucinous and non-mucinous colorectal adenocarcinoma patients based on their tumor locations, with details in treatment types, chemotherapy regimen, tumor stages, and number of patients recorded.

**Table 1:** Differences and comparison between mucinous and non-mucinous patients with colorectal cancer in 14 clinical trials

Clinical trial	Type of hemotherapy	Stage	Chemotherapy regimen	Patients (*n*, MC/NMC)	Median OS (months)		OS rate (%)		HR
					MC	NMC	MC	NMC	
*Tumor location: colorectal*									
Negri 2005 [63]	Palliative	IV	5-FU based first-line chemotherapy	135 (45/90)	11.8	17.9	–	–	1.50
Catalano 2009 [8]	Palliative	IV	5-FU with OXA and/or CPT-11 based first-line combination chemotherapy	255 (49/206)	14.0	23.4	53.1% (1-year)	77.4% (1-year)	1.59
Mekenkamp Study 1 2012 [64]	Palliative	IV	First-line sequential or combination treatment with CAP, CPT-11 and OXA.	485 (50/435)	13.2	19.2	–	–	1.80
Mekenkamp Study 2 2012 [64]	Palliative	IV	CAP, OXA and BEV with or without CET	525 (49/476)	13.1	21.5	–	–	1.76
Park 2015 [65]	Adjuvant	I–III	5-FU based chemotherapy	6475 (274/6201)	–	–	81.4% (5-year)	87.4% (5-year)	1.58
*Tumor location: colon*									
Maisano 2012 [60]	Palliative	IV	FOLFOX-4 regimen [OXA, LV, 5-FU]	63 (21/42)	8.0	18.0	–	–	1.99
Catalano 2012 [23]	Adjuvant	II–III	Fluoropyrimidine-based or OXA-based chemotherapy	1025	–	–	78.6% (5-year)	72.3% (5-year)	0.89
Kim 2013 [14]	Adjuvant chemotherapy	III	FOLFOX chemotherapy [LV, 5-FU, OXA]	394	–	–	56.9% (3-year DFS)	79.2% (3-year DFS)	1.82
*Tumor location: rectal*									
Sengul 2006 [66]	PCRT	III–IV	45–60 Gy and an infusion of 5-FU	46 (11/35)	–	–	–	–	–
Grillo-Ruggieri 2007 [98]	PCRT	II–IV	50.4 Gy and 5-FU-based chemotherapy	136 (25/111)	–	–	89.0% (5-year)	83.9% (5-year)	0.35
Shin 2011 [99]	PCRT	III–IV	45–50.4 Gy and 5-FU- or CAP-based chemotherapy	368 (23/345)	–	–	64.8% (5-year)	79.8% (5-year)	2.36
Simha 2014 [67]	PCRT	I–IV	45 Gy and 5-FU and LV	162 (34,128)	–	–	–	–	–
Hugen 2015 [69]	PCRT	II–III	45–50.4 Gy and CAP with or without OXA/BEV or 5-FU	540 (58/482)	–	–	53.1% (5-year)	54.1% (5-year)	–
Hugen 2013 [9]	Adjuvant	I–IV	5-FU or CAP with or without OXA	9045 (744/8301)	–	–	41.0% (5-year)	51.2% (5-year)	1.22

*MC* mucinous colorectal adenocarcinoma, *NMC* non-mucinous colorectal adenocarcinoma, *PRCT* preoperative chemoradiotherapy, *OS* overall survival, HR hazard ratio, *5-FU* 5-fluorouracil, *OXA* oxaliplatin, *CAP* capecitabine, *BEV* bevacizumab, *CET* cetuximab, *LV* leucovorin, *CPT-11* irinotecan

Five studies have investigated the chemotherapy effects on CRC patients, and each of them concluded that mucinous colorectal adenocarcinoma patients had a shorter median OS than non-mucinous colorectal adenocarcinoma patients despite different chemotherapy regimens being used in these trials. Negri et al. [63] administered 5-fluorouracil (5-FU)-based first-line chemotherapy to stage IV mucinous and non-mucinous colorectal adenocarcinoma patients as palliative care and found a median OS of 11.8 and 17.9 months, respectively [hazard ratio (HR), 1.50; 95% confidence interval (CI), 1.02–2.19; *P *= 0.037]. Catalano et al. [8] prescribed 5-FU with oxaliplatin (OXA) and/or irinotecan (CPT-11)-based first-line combination chemotherapy to stage IV mucinous and non-mucinous colorectal adenocarcinoma patients and found a median OS of 14.0 and 23.4 months, respectively (HR, 1.59; 95% CI 1.05–2.40; *P *= 0.027). Mekenkamp et al. [64] first applied first-line sequential or combination treatment with capecitabine (CAP), CPT-11 and OXA to 485 patients and found that the median OS was 13.2 and 19.2 months for patients with and without mucinous colorectal adenocarcinoma histology, respectively (HR, 1.80; 95% CI 1.24–2.62; *P* = 0.003). In patients receiving CAP, OXA and bevacizumab (BEV) with or without cetuximab (CET), the median OS was 13.1 and 21.5 months, respectively (HR, 1.76; 95% CI 1.16–2.67; *P* = 0.008) [64]. Further, Park et al. [65] showed that the 5-year OS was significantly lower (81.4%) for stage I, II, and III mucinous colorectal adenocarcinoma patients as compared to non-mucinous colorectal adenocarcinoma patients (87.4%, *P* = 0.005) when both groups were given adjuvant 5-FU based chemotherapy.

The treatment of colonic mucinous cancer was also investigated in one palliative study and two adjuvant studies. Maisano et al. [60] treated stage IV colonic cancer patients with the FOLFOX-4 (folinic acid, 5-FU and oxaliplatin) regimen and observed a median OS of 8.0 and 18.0 months in patients with and without mucinous colorectal adenocarcinoma histology, respectively (HR, 1.99; 95% CI 1.26–1.70; *P* = 0.03). In the study conducted by Catalano et al. [22], fluoropyrimidine-based or OXA-based chemotherapy was used as adjuvant chemotherapy in stage II/III colonic adenocarcinoma patients. The 5-year OS was 78.6% and 72.3% for the mucinous and non-mucinous colorectal carcinoma, respectively (HR, 0.89; 95% CI 0.59–1.69; *P* = 0.532). Kim et al. [14] administered the FOLFOX-4 regimen to stage III patients as adjuvant chemotherapy, and a 3-year disease-free survival (DFS) of 56.9% and 79.2% were observed in mucinous and non-mucinous colonic adenocarcinoma patients, respectively (HR, 1.82; 95% CI 1.03–3.23; *P* = 0.040). These findings show that the prognosis for mucinous colonic adenocarcinoma patients was generally poorer as compared to the patients with non-mucinous colonic adenocarcinoma.

Researches that focus on rectal mucinous cancer mainly studied the effect of preoperative chemoradiotherapy (PCRT). Four out of five studies showed that in rectal mucinous adenocarcinoma, the mucinous rectal adenocarcinoma histology served as a biomarker for poor prognosis after PCRT. Patients with rectal mucinous adenocarcinoma were more prone to have lower survival rates and poorer downstaging. Sengul et al. [66] demonstrated that after receiving preoperative irradiation and infusion with 5-FU, mucinous rectal adenocarcinoma patients at stage III and IV had a higher tumor regression grade and a smaller transrectal ultrasound score than non-mucinous cancer patients. Similar results were found by Simha et al. [67] in studies where mucinous and non-mucinous rectal adenocarcinoma patients were treated with preoperative radiation and 5-FU plus leucovorin chemotherapy. It was further shown that adjuvant chemotherapy after total mesorectal excision (TME) surgery served as an independent factor for improving prognosis for patients with mucinous rectal adenocarcinoma. Thus, it is advised that adjuvant chemotherapy should be offered to patients with rectal mucinous adenocarcinoma histology who have undergone TME surgery [68]. Further, a study by Hugen et al. [69], in which PCRT treatments or adjuvant chemotherapies were prescribed to patients with rectal adenocarcinoma, found that the gap in survival rates between mucinous and non-mucinous rectal adenocarcinoma could be narrowed when modern preoperative treatments such as preoperative short-term radiotherapy, preoperative chemoradiotherapy and TME surgery.

Chemotherapies including the FOLFOX-4, XELOX (capecitabine and oxaliplatin), and FOLFIRI (folinic acid, fluorouracil and irinotecan) regimen are considered as regular treatment options for mucinous colorectal adenocarcinoma patients. Monotherapy with 5-FU is also recommended for relatively frail patients (Eastern Cooperative Oncology Group Performance Status (ECOG PS) score 2–3) who are unable to continue combined therapy. Liu et al. [70] found that the FOLFIRI regimen could prolong PFS by 5 months compared with the FOLFOX-4 regimen for mucinous colorectal adenocarcinoma patients (*P* = 0.038), suggesting that the FOLFIRI regimen could be first considered for this group of patients.

### Targeted molecular therapy

Bevacizumab and cetuximab are two drugs that are commonly used in targeted molecular therapy for advanced CRC patients. Cetuximab is an anti-epidermal growth factor receptor (anti-EGFR) antibody and is often used in combination with chemotherapy. Currently, a small number of studies have investigated the treatment prognosis for cetuximab in mucinous and non-mucinous colorectal carcinoma patients with wild-type *KRAS*. Evidence suggested that for CRC patients with left-sided tumors, chemotherapy plus anti-EGFR antibody therapy demonstrated an enhanced outcome (HR, 0.75; 95% CI 0.67–0.84; *P* < 0.001), while the same regimen exhibited no significant benefit for CRC patients with right-sided tumors (HR, 1.12; 95% CI 0.87–1.45; *P* = 0.381) [71]. Venook et al. [72] showed that when the target tumor was located in the right colon, which is commonly found in mucinous colorectal adenocarcinoma, patients in the bevacizumab-chemotherapy group had a median OS of 24.5 months, while patients who received cetuximab-chemotherapy had a median OS of 16.4 months. Further, a nationwide population-based study indicated that the addition of bevacizumab to chemotherapy was associated with longer OS than palliative chemotherapy alone (HR, 0.7; 95% CI 0.64–0.83) in CRC patients with peritoneal metastasis [73]. However, no significant difference in OS was found in *KRAS* wild-type advanced or metastatic CRC patients between the addition of cetuximab versus bevacizumab to chemotherapy as a first-line treatment (HR, 0.88; 95% CI 0.77–1.01; *P* = 0.08) [74]. A study on ovarian mucinous adenocarcinoma showed that cetuximab inhibited the growth of tumor cell lines lacking *KRAS* mutation, but could not inhibit the growth of mucinous tumor cells carrying a mutation in *KRAS* gene [75]. Few comprehensive clinical studies have investigated the effects of targeted molecular therapy in patients with mucinous adenocarcinoma histology, but there were some which have been reported in mucinous ovarian carcinoma [75–79]. It was shown that a woman with a locally advanced mucinous colorectal adenocarcinoma in the transverse colon who was treated with 4 months of palliative metronomic capecitabine with bevacizumab, subsequently underwent radical surgery and the treatment intention was changed from palliative to curative. This evidence suggesting the potential use of targeted molecular therapy for mucinous colorectal adenocarcinoma patients [80]. In this regard, targeted molecular therapy is only recommended for stage IV metastatic CRC patients but not stage I/II/III postoperative patients, due to insufficient evidence in literature. Bevacizumab is advised for patients with right-sided tumors or *RAS*-mutations. For mucinous colorectal adenocarcinoma patients with left-sided tumors, bevacizumab could be used as a first-line of treatment, and cetuximab as a second-line of treatment but if the treatment fails, then regorafenib could be used as a third-line of treatment. Bevacizumab could also be considered as first-line treatment for CRC patients with peritoneal metastasis. As the tumors with mucinous colorectal adenocarcinoma histology are more likely to be located on the right side of the colon in patients with *KRAS* mutations, we suggest using bevacizumab as the first choice for mucinous colorectal adenocarcinoma.

### Hyperthermic intraperitoneal chemotherapy (HIPEC)

HIPEC is often used to eradicate microscopic residual disease, especially peritoneal dissemination of cancers. As previously mentioned, since a higher ratio of mucinous colorectal adenocarcinoma patients have peritoneal metastases as compared to non-mucinous colorectal adenocarcinoma patients, HIPEC can become an essential treatment option for such patients. A recommendation on a standardized delivery of HIPEC in patients with CRC was proposed in 2014 [81], which aimed to maximize the efficacy of seven key HIPEC parameters including method, inflow temperature, perfusate volume, drug, dosage, timing of delivery, and total perfusion time. Although a detailed plan for mucinous colorectal adenocarcinoma has not yet been well studied, the standard therapy which was delivered intraoperatively at the time of cytoreduction using a closed technique for peritoneal dissemination from CRC was also reported to be used in appendiceal primary tumors, and may be a potential therapeutic may further be explored for mucinous colorectal adenocarcinoma [82]. In addition, early postoperative intraperitoneal chemotherapy (EPIC) using floxuridine, Mitomycin C (MMC) or 5-FU are recommended as well [82]. HIPEC can thus recommended for mucinous colorectal adenocarcinoma patients with peritoneal metastases as a first-line therapy, although more comprehensive evaluation of the overall response rate and OS is further needed.

### Nanoparticle drugs

New targeted drugs for mucinous adenocarcinoma are under investigation [83, 84]. Mucus is a complex hydrogel covering the epithelial surfaces and forms a barrier to protect the underlying tissues from the extracellular environment, thereby adversely affecting the permeation and action of some drugs [85]. The pore size of the mucous layer is approximately 100 nm to 200 nm. Thus, only nanoparticles could possibly penetrate these layers and reach the targeted tissues [86]. Solubility and lipophilicity are both critical for drug absorption, since high solubility ensures drugs to be dissolved in body fluids while high lipophilicity ensures drugs to permeate the biological membrane, thus, poorly water-soluble lipophilic drugs combined with cyclodextrins are the best option for forming water-soluble complexes that possess high permeability through lipophilic membranes [87]. Moreover, feasible strategies to improve drug oral bioavailability include the addition of a coating of polymer molecules to help the nanoparticles sneak through the net and a nanoparticle carrier for exploiting the mucolytic agents that are able to cleave the glycoprotein substructures of mucus [83, 88]. Albumin-bound paclitaxel is commonly used as the regimen of nanodrug treatment in breast, pancreatic, and lung cancer. Despite the promising effect of nanodrugs in mucinous adenocarcinoma [35], no clinical trials have been conducted to evaluate its safety, overall response rate, and OS. Such trials are thus urgently needed before nanodrugs could be used for mucinous adenocarcinoma patients.

### Immunotherapy

The programmed cell death protein 1 (PD-1) is triggered by its ligand, programmed cell death ligand 1 (PD-L1), to inhibit T cell activation and consequently hampering the host immune response against cancer cells. Blockade of this pathway by inhibitors of PD-1 or PD-L1 has led to significantly improved clinical outcomes in many types of cancer including melanoma, non-small-cell lung cancer, and renal cell cancer [89]. Tumors displaying mismatch repair deficiency (dMMR) are distinguished from mismatch repair-proficient ones for their high expression of checkpoint molecules, including PD-1, PD-L1, cytotoxic T-lymphocyte-associated protein 4 (CTLA-4), lymphocyte activation gene 3 (LAG-3) and indoleamine 2,3-dioxygenase (IDO), whose immune microenvironment is targeted and balanced by inhibitors that resist tumor elimination [90].

Mucinous colorectal adenocarcinoma is associated with a higher rate of MSI-H. It has been proven that cancer patients with MMR/MSI-H tumors are more likely to benefit from anti-PD-1 therapy, and similar results were observed in MSI CRC patients with 31.3% patients achieving an investigator-assessed objective response and 69% patients having disease control for 12 weeks or longer [90–92]. MSI CRC only accounts for about 15.0% cases of all CRC, yet the fact that mucinous colorectal adenocarcinoma is associated with higher rate of MSI-H implied mucinous colorectal adenocarcinoma as a good candidate for PD-1 inhibitor treatment. However, Kim et al. [93] suggested that in MSI-H CRC, the presence of mucinous colorectal adenocarcinoma was associated with a poorer response to PD-L1 as compared to patients lacking the mucinous component.

Nevertheless, multiple combination therapies have proven to enhance the clinical outcomes of CRC patients, though specific data were lacking in the subgroup of mucinous colorectal carcinoma patients. Among dMMR/MSI-H CRC patients, 77% had reduced tumor burden from baseline; 76% and 87% of the patients showed a 9-month PFS with nivolumab (NIVO) monotherapy and NIVO in combination with ipilimumab (IPI) therapy, respectively, suggesting an enhanced clinical benefit and manageable safety for NIVO and IPI [94]. More recently, NIVO and low-dose IPI was given to CRC patients with MMR/MSI-H as a first-line therapy, which demonstrated a robust and durable clinical benefit with the objective response rate (ORR) of 60% and the disease control rate of 84%; suggesting this immunotherapy regimen as a first-line treatment option for CRC patients [95]. Furthermore, a median PFS of 11.7 months with atezolizumab in combination with bevacizumab versus 8.4 months with sunitinib was observed in renal cell carcinoma patients [96]. The first study of a PD-1 inhibitor and VEGF blockade in MSI-H CRC also reported a 90% disease control rate and 30% ORR with further follow-up ongoing, suggesting a possible role of anti-VEGF in enhancing antitumor activity in immune checkpoint blockade [97].

## Conclusion

This review summarized the current research progress in genetic alterations of mucinous colorectal adenocarcinoma and brought forward the current treatment options for these patients. Although the exact mechanism that leads to extensive mucin in tumors is not yet well understood, genetic aberrations could be the potential reasons that determine the oncogenesis and survival differences between mucinous colorectal adenocarcinoma and other subtypes of CRC.

The effects of palliative chemotherapy in mucinous and non-mucinous colorectal adenocarcinoma patients were also reviewed. Clinical studies comparing the OS of colonic cancer patients suggest that the mucinous colorectal adenocarcinoma histology was an adverse prognostic indicator. For rectal patients, on the other hand, contradictive results were found in studies concerning the effects of PCRT between these two groups of patients. In this regard, the prognosis of mucinous as to non-mucinous colorectal adenocarcinoma is still controversial, although the OS of mucinous colorectal adenocarcinoma patients tended to be poorer than non-mucinous colorectal adenocarcinoma patients in the majority of studies. These further warrant more focus for therapies carefully tailored to mucinous colorectal adenocarcinoma patients based on their genetic alterations.

Moreover, future prospective clinical trials on the effect of nanodrugs, targeted therapy, and immunotherapy should be conducted on mucinous colorectal adenocarcinoma patients to evaluate their safety and efficacy in this specific group of patients. In addition, studies based on tumor location would help to address why distinct responses to treatments have been found in clinical practice. The development and implementation of treatment guidelines for mucinous colorectal adenocarcinoma patients would further lead to improved survival and better outcomes for these patients.

## Abbreviations

CRC: colorectal cancer
MSI: microsatellite instability
PFS: progression-free survival
CIMP: CpG island methylator phenotype
MUC2: mucin-2
MUC5AC: mucin 5AC
MUC5B: mucin 5B
MUC6: mucin 6
SOX2: SRY (sex determining region Y)-box 2
MLH1: MutL homolog 1
MSH2: MutS protein homolog 2
MSH6: MutS protein homolog 6
KRAS: v-Ki-ras2
BRAF: B-Raf proto-oncogene
OS: overall survival
MMR: mismatch repair
VEGF: vascular endothelial growth factor
MRI: magnetic resonance imaging
CT: computed tomography
HR: hazard ratio
CI: confidence interval
5-FU: 5-fluorouracil
OXA: oxaliplatin
CPT-11: irinotecan
BEV: bevacizumab
CET: cetuximab
FOLFOX: folinic acid, 5-FU and oxaliplatin
XELOX: capecitabine and oxaliplatin
FOLFIRI: folinic acid, fluorouracil and irinotecan
ECOG PS score: Eastern Cooperative Oncology Group Performance Status
PRCT: preoperative chemoradiotherapy
TME: total mesorectal excision
anti-EGFR: anti-epidermal growth factor receptor
HIPEC: hyperthermic intraperitoneal chemotherapy
EPIC: early postoperative intraperitoneal chemotherapy
MMC: mitomycin C
PD-1: programmed cell death receptor
PD-L1: programmed death-ligand 1
CTLA-4: cytotoxic T-lymphocyte-associated protein 4
LAG-3: lymphocyte activation gene 3
IDO: indoleamine 2,3-dioxygenase
NIVO: nivolumab
IPI: ipilimumab
dMMR: mismatch repair deficiency
ORR: objective response rate
